# Supplementary material for: DArTseq-based silicoDArT and SNP markers reveal the genetic diversity and population structure of Kenyan cashew (Anacardium occidentale L.) landraces
Source: PLoS One. 2025 Jan 31;20(1):e0313850. doi: 10.1371/journal.pone.0313850 (PMC11785325; doi:10.1371/journal.pone.0313850)
Supplement: Attachment — Submitted filename: Point-by-point response.docx [file pone.0313850.s004.docx]

Point-by-point response

| **Reviewer** | **Reviewers comment** | **Authors’ Response** |
| --- | --- | --- |
| Reviewer #1 | Revi |  |
|  | lthough the research work done in this study is good, but introduction and conclusion needs to be improved. | The introduction and conclusions have been rewritten |
|  | I suggest author to include AMOVA for study the diversity among the populations and within the populations. They should also put the reason behind the low diversity among the Cashewnut cultivar in Keny | We have included AMOVA in the resubmitted manuscript pls FST |
|  | The conclusion should be strong and clearly suggesting the breeders how they can improve the cultivar so that the yield and health of plant can be improved. | We have rewritten the conclusion in include the suggested comment |
| Reviewer #2 | The language of the manuscript is ok and the terminology is suitable. | We have rewritten the whole manuscript to make it readable |
|  | The dendrogram of for the genetic dissimilarity is not clear and there is not much more information about the kinship relations of the cashew genotypes. Unfortunately, there is no information about the genotypes, we just know they are collected from three counties | We collected landraces. The denogram are now clear. |
|  | There should be a table about the genotypes, which might be useful to understand the finding and related results for readers and for future users of the outputs. | We collected landraces and do not have prior information of them |
|  | In the conclusion section it is concluded that “breeders can make informed decisions for parent selection, potentially improving traits like yield, resistance to pests, and adaptability” how it is possible to make decisions for yield, resistance to pest and adaptability from this data presented in the manuscri | We have rewritten the conclusion to include the reviewer’s comment |
