# Supplementary material for: DArTseq-based silicoDArT and SNP markers reveal the genetic diversity and population structure of Kenyan cashew (Anacardium occidentale L.) landraces
Source: PLoS One. 2025 Jan 31;20(1):e0313850. doi: 10.1371/journal.pone.0313850 (PMC11785325; doi:10.1371/journal.pone.0313850)
Supplement: Attachment — Submitted filename: PONE-D-24-27021_review.docx [file pone.0313850.s005.docx]

Overall

The manuscript on the ‘Genetic diversity and population structure of Kenyan cashew (Anacardium occidentale L) landraces using DArTseq-based silicoDArT and SNP markers’ has a strong scientific merit. The genetic resources from the study are important for breeding and conservation. However, some of the results and discussion are still not very clear and need revision. The Authors should also consider a language revision.

The point-to-point comments are as follows;

P3, L2-3; please clarify the statement. We know that most trees provide the services mentioned in L1-2. Is there any specific feature exhibited by cashew to suggest any adaptive advantage of this species in providing the services mentioned?

P3, L3-5; this looks like a general statement when it comes to tree technologies so, please explain further how cashew specifically can support climate-smart technologies or climate change adaptation

P3, L9; add a coma after East Africa

P3, L15-16; the Authors mention that the flowers are self-pollinated and at the same time allogamous. This is very contradictory, please check

P4, L2; delete hew

P4, L12; add a comma after DaRT

P4, L13-17; revise statement for clarity

P5, L1-2; Split into two sentences for clarity, replace ‘over’ with ‘in’ not to be repetitive. Therefore, I suggest; DArT is a popular platform for genomic research because it produces many molecular markers over the entire genome in a short period. It is also straightforward to use and reasonably cost-effective (Deres and & Feyissa, 2022).

P5, L7 Please refer to this publication for your information; Discovery Of Novel Single Nucleotide Polymorphic (Snp) Markers For Genetic Mapping Of Cashew (*Anacardium Occidentale*. L)
P5, L16 replace ‘leaves’ with ‘leaf’

P5, L17 delete extra bracket from citation

P5, L18 Add a comma after strata

P5, L17-20 clarify on the proportional bit of the trees and the farmers. What were the sizes per population? This is important since it has a bearing on the allelic richness estimates.

P5, L16-23 needs to be streamlined to avoid repetitions

P7, L4, indexes or indices?

P7, L14 replace ‘plotted’ with ’generated’

P7, L22 add a comma after filtered

P8, L1 delete the p and unify the decimal places

P8, L4 the SNP-based expected heterozygosity range is 0.33?

P8, L7-9 I don’t see how the cross-entropy or scree plots identify the two clusters. These need further explanation.

P8, L14 I suppose the values in brackets are FST.

P8, L14 an FST of 0.005 or even 0.01 are not high to say there is divergence. It is even clear that clusters are not delineated based on these predefined sub-counties. What about differentiation between Kwale and Kalifu?

P8, L16-18 This sentence is not clear, please rewrite. Also, I don’t see any differentiation between Kilifi and Lamu given the very low FST. You need to test the significance of the clusters shown on Figure 4 especially given the very low FST values among these populations

P8, L16 PCoA not PCA

P8, L20-22 The roots of the dendrogram of for the genetic dissimilarity are not clear to decipher any relationships. I see four colour clusters on the dendrogram. Please clarify. Also clarify the 2 subpopulations based on K- analysis and 3 clusters based on PCoA and dendrogram.

P9, L10, change ‘differentiation’ to ‘delineation’ to reduce confusion

P9, L11 What are these ‘conventional molecular markers’?

P9, L15 Provide the average PIC. This, not the range would give a better picture of the level of heterozygosity

P9, L19, replace ‘between’ with ‘among’

P10, L1 please explain how domestication and distribution affect genetic diversity

P10, L6-13, the text is unclear and needs revision

P10, L14, the allelic richness should be discussed in view of population sizes.

P11, L3, in this case what is artificial hybridization? Are there occurrences of controlled crossing?

P11, L4-5 contradicts the results on P8, L13-14. Please reconcile

P11, L7 there is no genetic differentiation across clusters, so please clarify how selecting individuals in these clusters can increase genetic gain.

P11, L11 reconcile with the 2 subpopulations, on P8 L7-8

P12, L11 2, 3 or 4 subpopulations?

Citations need to be consistent, with DOIs
