# Supplementary material for: DArTseq-based silicoDArT and SNP markers reveal the genetic diversity and population structure of Kenyan cashew (Anacardium occidentale L.) landraces
Source: PLoS One. 2025 Jan 31;20(1):e0313850. doi: 10.1371/journal.pone.0313850 (PMC11785325; doi:10.1371/journal.pone.0313850)
Supplement: Attachment — Submitted filename: Response to Reviewers Cashew diversity_29.8.2024.docx [file pone.0313850.s007.docx]

| **Reviewer** | **Comment** | **Authors response** |
| --- | --- | --- |
| Reviewer #1  **Reviewer #2** | P3, L2-3. please clarify the statement. We know that most trees provide the services mentioned in L1-2. Is there any specific feature exhibited by cashew to suggest any adaptive advantage of this species in providing the services mentioned?  P3, L3-5. this looks like a general statement when it comes to tree technologies so, please explain further how cashew specifically can support climate-smart technologies or climate change adaptation  P3, L15-16. the Authors mention that the flowers are self-pollinated and at the same time allogamous. This is very contradictory, please check  P5, L7. Please refer to this publication for your information; Discovery of Novel Single Nucleotide Polymorphic (Snp) Markers For Genetic Mapping Of Cashew (*Anacardium Occidentale*. L)  P5, L17-20. Clarify on the proportional bit of the trees and the farmers. What were the sizes per population? This is important since it has a bearing on the allelic richness estimates.  P8, L4. The SNP-based expected heterozygosity range is 0.33?  P8, L7-9. I don’t see how the cross-entropy or scree plots identify the two clusters. These need further explanation  P8, L14. I suppose the values in brackets are FST.  P8, L14. An FST of 0.005 or even 0.01 are not high to say there is divergence  P8, L16-18. This sentence is not clear, please rewrite. Also, I don’t see any differentiation between Kilifi and Lamu given the very low FST. You need to test the significance of the clusters shown on Figure 4 especially given the very low FST values among these populations  P8, L20-22. The roots of the dendrogram of for the genetic dissimilarity are not clear to decipher any relationships. I see four colour clusters on the dendrogram. Please clarify. Also clarify the 2 subpopulations based on K- analysis and 3 clusters based on PCoA and dendrogram.  P9, L11. What are these ‘conventional molecular markers’?  P10, L1. P10, L1 please explain how domestication and distribution affect genetic diversity  P10, L14. the allelic richness should be discussed in view of population sizes. | P3, L2-3. This is a potential adaptation strategy to sub-optimal or adverse environmental conditions such as extreme salinity, poor drainage. Cashew plant has proofed effective since its inception to east Africa in 16^th^ century (Monteiro et al., 2015).  P3, L3-5. Additionally, cashews deep rooting system and its integration into agroforestry systems makes suitable for upscaling climate-smart technologies and building resilience to climate change among smallholder farmers.  P3, L15-16. Corrected  P5, L7. Got the correlation  P5, L17-20. All the plant materials in this study were landraces, and a total of 92 apical leaf samples (27 from Kwale, 28 Lamu, and 37 kilifi) were collected.  Yes, the value was constant across all therefore I did write it without showing the range.  This has been clarified in the main document and results section due to the repetition that was therein.  Yes they are.  Yes and this has been exaplained in revised manuscript.  The differentiation due to FST value among the study sites has been rewritten and its significance to cashew genomic breeding programs explained.  The genetic dendogram created using weighted neighbor-joining alogrithms in this study aimed at genetic structure understanding and diversity of the clusters. For both markers these two components have been outlined. The cross entropy criteria model is always best explained least value, thus in this study k value of 2 reflects best explanation by the presence of two major ancestral populations or genetic clusters. The PCoA plot underscores this where on the plot we have two clear distinct clusters. The color codes for Kilifi, Kwale and Lamu were used to help identify sample origins and therefore they are not regarded as distinct clusters.  These are specific sequences of DNA that are used to identify and track genetic differences within and between populations, species, or individuals i.e **Restriction Fragment Length Polymorphisms (RFLPs),**  Simple Sequence Repeats (SSRs) or Microsatellites, Random Amplified Polymorphic DNA (RAPD) e.t.c.  Domestication promotes homogeneity which limits heterozygosity rigor among species thus making a population less diverse genetically.  This has been addressed in p12 L21-23 and p13 L1-L6. |
|  | **Abstract**  Page 1 L 14 – 21: this background is too long, it should be summarized to one sentence and focus on the aim and short description of the methods.  Page 1 L 18: the authors indicated that several techniques have been employed to know the genetic diversity of different plants. It would be better they indicate whether there are some techniques used specifically for Cashew plant.  Missing quality criteria parameters (call rate and reproducibility).  Clarify study aim.  The authors should also revise the conclusion and relate it to the topic of the manuscript.  **The section of materials and methods**  The sampling method was not clearly described, it lacks the names of sub-counties, and the number of cashew landraces (samples) collected from each subcounty and each county.  What is the motivation behind the choice of limiting the study to three counties (Kwale, Kilfi and Lamu).  Clarify the use of PIC as genetic diversity index.  Clarify on how population structure was determined.  **Results Section**  Elaborate the key components in the topic such as genetic diversity and population structure.  some results presented in this section do not match those presented in Table 2.  These findings (He and Ho) were also presented in no right subsection “markers’ characterisation” and are not the same as those presented in Table 2  The authors should also explain why no genetic indices of cashew landraces from Lamu were calculated  **Discussion Section**  The authors stated that the expected heterozygosity was greater than the observed heterozygosity in contrast of what presented in Table 2 (which misses the indices of Lamu population).  The authors should justify thoroughly their confirmation without ambiguity that the moderate allele richness observed in cashew landraces explains their ability to adapt to both diseases and climate changes.  **Conclusion section**  Conclusions should be drawn by also considering the key findings such as relationship between landraces (ancestral subpopulations and three subpopulations), admixture and the potential fixation of alleles revealed by the heterozygosity.  Authors should also highlight some limitations and suggest or propose the potential future research. | This has been corrected pg 1 L19-L23 and pg2 L1-2.  This has been clarified pg1 L21-L23.  This is included from pg2 L15-L16.  This is clarified in pg2 L22-L23.  Pg6 L22-L23 and pg7 L1-L5, this has been clarified including citation of a table.  Kilifi, Kwale and Lamu are three major cashew producing counties dating back as from 1980s which was considered a major cash crop as documented by KALRO (Kenya Agricultural and Livestock Research Organization). However as of date cash production has declined across all counties with some region experiencing massive cutting down of cashew trees for firewood etc.  Pg 8 L18-L22 and pg 9 L1-L8 has outlined the determination of cashew landraces structure.  Elaborated in Pg 10 L8-L9, and L12-15.  This has been counter checked and corrected accordingly.  This has been reshuffled and rearranged in correct order.  This was a mistaken omission however we have included them.  From pg12 L15-L23 we have explained the significance of Ho>He.  From pg 13 L1-L9 has outlined the influence of the moderate allele richness noted in the study findings.  This has been addressed as from pg 17 L12-L22. |
| **Reviewer #3** | **Abstract.**  **Introduction**  Background Information- provide more context on the importance of genetic diversity studies in cashew could strengthen the rationale.  **Current Knowledge Gaps-** Clearly state the gaps in current research, specifically what this study addresses, to provide a better foundation for the objectives.  **Objectives**: The objectives are implicit. It would be helpful to explicitly state the primary goals of the study.  **Materials and Methods**  **Sampling Strategy**: The sampling strategy is described, but more detail on the stratified random system and why it was chosen would enhance clarity.  **DNA Extraction**: The DNA extraction method is standard, but mentioning any modifications or challenges encountered during the process could be useful for reproducibility.  **DArTseq Genotyping**: This section is detailed but could benefit from a flowchart or diagram to visually represent the workflow from DNA extraction to genotyping.  **Statistical Analysis**: The methods for statistical analysis are appropriate. However, providing more detail on the software versions and specific packages used in R would improve transparency  **Results**  **Data Presentation**: The presentation of results is clear, but the inclusion of more figures, such as heatmaps or additional PCA plots, could help visualize the data better.  **Marker Quality**: The discussion on marker quality is adequate. Adding comparative analysis with other studies using similar markers would contextualize the findings.  **Genetic Relationships**: The results on genetic relationships and population structure are significant. However, discussing potential reasons for the observed admixture and gene flow in more detail would add depth.  **Discussion**  **Interpretation of Findings**: The discussion interprets the results well, but linking the findings to practical implications for breeding programs could be elaborated.  **Comparative Analysis**: Compare the results more extensively with similar studies in cashew or other crops to highlight the significance and novelty of the findings.  **Limitations**: Address the limitations of the study, such as the sample size or geographical constraints, and how they might affect the results.  **Conclusion**  **Summary of Findings**: The conclusion summarizes the findings effectively. However, suggesting specific future research directions based on the results would enhance this section.  **Practical Implications**: Highlight the practical implications for cashew breeding programs and conservation efforts more explicitly.  **Figures and Tables**  **Figure Clarity**: Ensure all figures are high resolution and clearly labeled. Figures 2 and 3, depicting population structure and admixture, should have clear legends and be easy to interpret.  **Table Completeness**: Tables should be self-explanatory with detailed captions. Ensure all abbreviations are defined in the table legends. | This has been clarified in pg1 L-19-23.  Outlined in pg 1 L21-23.  Outlined in pg1 L23 and pg2 L1-2.  Stratified random sampling in this study was a major strength due to the fact that across the three study sites, the sub counties were ideal stratus to sample from given our study was conducted across the counties not distinct farms. More outline is in pg7 L1-5.  This is ideal however concerning this work, we reported what we carried out.  This is so ideal however looking at the outline made concerning the approach we feel its satisfactory in this regard however for presentation purpose such work flow is very useful.  The package versions has been provided.  Additional results are useful however in our regard we agreed to use these results.  This has been added.  Further discussion has been exhausted in reference to admixture and gene flow.  The link of our findings to practical approaches has been deduced pg12 L8-13, pg13 L6-8.  Our work has a strong comparison with Geradina Pantaleo Mzena (2018) work on cashews in Tanzania. Pg13, L11-17  Having conducted our sample collection across the three counties and considered sub counties as our stratus may limit our work due to uncontrolled exposure unlike in a specific orchard. However, this due to nature of cashew farming practice being carried in Kenya.  This has been outlined in pg17, L17-18.  Outlined in pg17, L20-22.  Corrected.  Corrected. |

**Authors responses to Reviewer’s comments**
