# Supplementary material for: DArTseq-based silicoDArT and SNP markers reveal the genetic diversity and population structure of Kenyan cashew (Anacardium occidentale L.) landraces
Source: PLoS One. 2025 Jan 31;20(1):e0313850. doi: 10.1371/journal.pone.0313850 (PMC11785325; doi:10.1371/journal.pone.0313850)
Supplement: Attachment — Submitted filename: renamed_9c41f.docx [file pone.0313850.s009.docx]

**Authors’ responses to reviewer’s comments**

| **Reviewers 1 comments** | **Responses** |
| --- | --- |
| However, (i) the abstract needs to be more representative of the entire manuscript by adding more results such as the fixation indices, clustering, etc. | This has been included “The silicoDArT and SNP markers had mean PIC values ranging from 0.02- 0.50 and 0.0- 0.5, with an allelic richness ranging from 1.992 to 1.994 for silicoDArT and 1.862 to 1.889 for SNP markers. The observed heterozygosity and expected values ranged from 0.50-0.55 and 0.34-0.37, and 0.56-0.57 and 0.33 for both silicoDArT and SNP markers respectively.” |
| P5, L17 ‘samples collected was proportional to the number of farmers and the density of cashew trees. Please clarify for example, did you collect samples from each farmer that had cashew trees? Did you sample all the trees that individual farmers had or what does proportionality mean here? | The sample collections were based on strata within the sample site. The proportionality therein backtraces on aspect of the cashew plants available during collection, however, does not mean all trees present were collected. Cashew farming systems in the three counties were mostly among old orchards. |
| P10, L1-2, please further explain how domestication relates to the genetic diversity. Ideally most domestication events are supposed to reduce diversity due to founder events and genetic bottlenecks associated with the sampling process. | Absolutely right. Domestication influences genetic diversity and can result in diversity loss /reduction.  The authors have responded and made an alignment for consistency and coherence. |
| P10, L7-10, heterozygote advantage may not be quite right here. Outcrossing is an OK explanation, and possibly there could have been more than one domestication events that caused mixing of two previously isolated populations as suggested on P11, L18-24. | This has been considered and outcrossing is a key drive considering most of our cashew landraces used in this study are for decades thus the chance of crossing mixing is a factor to consider. |
| P11, L7-8 The fixation indices are too low, and this should suggest high gene flow. Please revise the controversies here. The low fixation indices explain the lack of distinct population structure suggested in L9. | This has been aligned and its an impact of domestication hence its fixation indices are low across all the study sites and the overall FST value. |
| P12, L12-14, could you please explain the 3 clusters vs K=2 | The selection of K = 2 effectively captures these regional differences in genetic structure and from the model the identification of the two sub populations is indicates a pattern of similarity among cashew landraces from Kwale, Lamu and Kilifi hence they share a genetic structure with an even distribution of two ancestral populations. The dendrogram clusters which revealed three clusters is a genetic variation of all samples and this evident in clustering both in silicoDArT and SNP markers |
| Fig 4 and 5, Could you please color the different sub-populations differently for easy visualization of admixing. | The admixture barplot generated two distinct clusters hence we sorted the isolates based on geographical locations and coded them in rationale to clustering results. However we are concerned that it may not be feasible to color code geographical location and clustering simultaneously as makes the visualization tainted. |
