# Supplementary material for: DArTseq-based silicoDArT and SNP markers reveal the genetic diversity and population structure of Kenyan cashew (Anacardium occidentale L.) landraces
Source: PLoS One. 2025 Jan 31;20(1):e0313850. doi: 10.1371/journal.pone.0313850 (PMC11785325; doi:10.1371/journal.pone.0313850)
Supplement: Attachment — Submitted filename: Response to Reviewers_7.10.2024.docx [file pone.0313850.s011.docx]

**Authors’ responses to reviewer’s comments**

| **Reviewer’s comment** | **Authors response** |
| --- | --- |
| P1, L20 Remove extra dot after resources | Corrected as directed |
| P2, L11-12, To make the sentence clear, I suggest to delete ‘thereby optimizing the effectiveness of selection’ | Corrected as advised |
| P3, L7-12, I suggest these edits for clarity…Cashew trees play key biodiversity roles as sources of food and shelter for wildlife. Additionally, the cashew tree deep rooting system supports the provision of ecosystem services such as soil stabilization, erosion prevention and carbon sequestration [7]. This is a potential adaptation strategy to suboptimal or adverse environmental conditions such as extreme salinity and poor drainage suggesting that its integration into agroforestry systems makes it suitable for upscaling climate-smart technologies and building resilience to climate change among smallholder farmers. | Revised as advised |
| P3, L19-22 needs a citation | Citation added |
| P4, L9 add a comma after DaRT | Revised as advised |
| P4, L12-13, revise sentence to reduce repetitions of words. I suggest you delete ‘in cashew’ at the end of the sentence. Add a comma after genomic studies | Revised as advised |
| P4, L23, a citation is not numbered, please check | This was corrected |
| P6, L15, a citation is not numbered, please check | This was corrected and all citations were renumbered |
| P6, L17, a citation is not numbered, please check | This was corrected |
| P7, L1, package v.3.19 were, change ‘were’ to ‘was’ | Revised as advised |
| P8, L9-11, For clarity, I suggest an edit to the sentences from ‘The overall fixation indices among the subpopulations for the silicoDArT and SNP 10 markers were 0.003 and 0.008, respectively (Table 4). Additionally, the extent of genetic differentiation across the three study sites was substantially low (Table 4)’ to ‘The extent of genetic differentiation across the three study sites was substantially low (Table 4) as indicated by the low overall fixation indices among the subpopulations for both the silicoDArT (Fst = 0.003) and SNP (Fst = 0.008) markers (Table 4)’. | Revised as advised |
| P9, L5 delete ‘grain’ | Revised as advised |
| P9, L6, I suggest you change magnitude’ to ‘efficiency’ | Revised as advised |
| P9, L16, put citation after genomic analysis | Citation inserted |
| P9, L18, a citation is not numbered, please check | Citation numbered |
| P10, L3-4, I again would suggest to delete ‘heterozygote advantage’ for clarity. | Revised as advised |
| P10, L8, if they are detrimental, change ‘are’ to ‘might/could’ | Revised by using “could be”as advised |
| P10, L8-10, Delete sentence as it doesn’t give additional information to the paragraph | Sentence deleted. |
| P11, L1-2 The low fixation indices suggest **HIGH** not ‘limited’ gene exchange  between populations, | Revised as advised |
| P11, L9.. Our study provides insight for evaluating genetic diversity and population structure about k=2, I SUGGEST you edit to Our study provides insight on population structure, where two clusters (k=2) were identified….. | Revised as advised |
| P12, L7 Revise sentence. | The sentence was revised to “Individual cashew landraces were clustered into three subpopulations, which correlated with their geographical locations. This observation supports the single domestication theory [39], highlighting a significant genetic bottleneck and limited genetic variation in cashew”. |
| P12, L11, delete grain | Replaced grain with nut |
